# The Local Awareness-Raising of Public Mental Health in the Capital City of Poland through Launch of Local Alliance against Depression

**DOI:** 10.3390/ijerph20053892

**Published:** 2023-02-22

**Authors:** Piotr Toczyski

**Affiliations:** Social Sciences, Maria Grzegorzewska University, 02-353 Warszawa, Poland; ptoczyski@aps.edu.pl

**Keywords:** depressive disorders, participatory action research, implementation science, 4-level intervention concept, action media, public mental health, radio broadcasting, digital media, local events, EAAD

## Abstract

The goal of this brief report is to show the specificity of building local alliances against depression in an Eastern European country within and after the series of 2021 lockdowns. It will be described in the form of a short communication piece. The semi-peripheral specificity of Poland adds some insights which will be useful for other leaders of similar alliances globally. This short report can be read as a higher resolution of the European Alliance Against Depression (EAAD) method activities known from other recent works. We want to answer the question of how to begin the activity and launch such an alliance in the semi-peripheral context of non-Western Europe.

## 1. Introduction

Depressive symptoms in the Polish population have been measured pre- and post-COVID-19 with PHQ-9 screening, leading to the conclusion that they may have risen to ca. 20% in the general population (https://pulsmedycyny.pl/cbos-co-piaty-polak-wykazuje-objawy-depresji-1155382, accessed on 15 February 2023). One of the WHO and European Commission Best Practice community interventions for public mental health are local alliances against depression. In Poland, one of the Eastern European countries that joined the EU in 2004, no local alliance against depression existed until 2021. The first one has been built since April 2021 within the framework of participatory action research. We are acting with the support of the capital city and district mayors, who have become allies of the district alliance. The opening of the alliance in Warsaw was held under the patronage of the European Commission Representation in Poland, and we were also a co-founder of the World Health Organization’s Pan-European Mental Health Coalition at the time of the opening ceremony. Within this framework, we are developing models of leadership in the field of public mental health, sharing the Warsaw experience with WHO stakeholders. We sensitize residents and institutions to the situation of people at risk of and experiencing depression. To this end, we are creating an inner-city alliance against depression, which is also intended to help prevent suicidal behavior. Formal and informal allies in this alliance become those individuals, institutions, and media interested in Warsaw who want to promote the three thoughts: depression can affect anyone; depression is a real disorder; and depression can be effectively treated.

### 1.1. Four-Level Intervention Concept (EAAD)

Among the most promising interventions for public mental health and suicide risk decrease, the alliance against depression method was recently named among the most promising [1,2,3,4,5,6,7]. In Poland, some of the earliest intuitions leading toward similar conclusions were once expressed by an outstanding individual: the rural doctor who moved his interests toward urban school medicine, counseling and suicidology. As he noticed in his unpublished government-commissioned report in 1970s and reminded in his public intellectual activities [8], the community-related tandem of the dysfunctional school environment and family home co-determined the depression, hopelessness, and desire to leave among adolescents in Poland. After 2000, in Europe, the 4-level approach was named Best Practice by the European Commission and WHO [3,9,10]. It has been implemented worldwide, especially in Europe [11,12,13]. The first Polish alliance against depression was launched as late as 2021. 

The four levels of the local alliance are as follows. On level one, primary care and mental health care are activated. Family doctors learn how to recognize and treat depression and explore suicidal tendencies [14]. On level two, the general public receives awareness campaigns. The public is addressed by large-scale campaigns, including information leaflets, brochures, and public events. The knowledge about depression treatment increases, and the stigmatization of depression is reduced [15]. On the third level, the community facilitators and stakeholders are targeted and educated to disseminate knowledge about depression Parents, youth workers, and teachers learn how to reach adolescents suffering from depression, self-harm, and suicidal behavior. Information sessions and prevention programs in schools are followed by close co-operation with the media to induce public discussion. Finally, on the fourth level, the patients, high-risk groups, and relatives receive direct access to professional help in a suicidal crisis. Anyone interested in starting a local or hyperlocal alliance may begin it themselves. The method is open to interested individuals.

### 1.2. The Best Practice Implementation Launched

The intervention concept on which the current intervention is based was the winner of the European Health Forum Award (5 October 2007). It has also been listed as Best Practice in several sources: the EC Green Paper on Mental Health (17 October 2005), a follow-up to the WHO Ministerial Conference on Mental Health (January 2005); the WHO report “Preventing suicide: A global imperative” (2014); and the EU-Compass for Action on Mental Health and Well-Being (2017). The community intervention for public mental health seen as both the EC and WHO Best Practice are local alliances against depression (AAD) founded according to the European Alliance Against Depression (EAAD) method. In Poland, one of the Eastern European countries that joined the EU in 2004, no local alliance against depression existed until 2021. The first one was built in April 2021 within the framework of participatory action research, and a year later, it officially opened, engaging four levels of the local community.

### 1.3. Best Practices for Public Mental Health

In 2008 the European Alliance Against Depression (EAAD) was founded by eight researchers from different universities, organizations, and institutions in Austria (Gesellschaft für Psychische Gesundheit–Pro mente Tirol), Belgium (LUCAS, Katholieke Universiteit Leuven), Germany (German Alliance Against Depression), Ireland (National Suicide Research Foundation), Netherlands (Trimbos Institute), Portugal (Clinica Universitaria de Psiquiatria e Saude Mental, Faculdade de Ciencias Medicas de Lisboa), Spain (Institut de Recerca, Hospital de la Santa Creu i Sant Pau) and United Kingdom (University of Stirling, Department of Applied Social Science). It now consists of 20 national chapters and 12 associate members in Europe, Australia, Canada, and South America.

The 4-level approach of EAAD to public mental health interventions has been implemented in different regions in 17 European countries comprising the four levels. The first is primary care and mental health care: educational workshops on how to recognize and treat depression and explore suicidal tendencies in the primary care setting. The second is the general public, at which large-scale public awareness campaigns (including posters, cinema spots, information leaflets, and public events) are targeted. The aim is to reduce the stigmatization of depression. The third level refers to community facilitators and stakeholders, who are approached with educational workshops and triggering public discussion. The fourth level is patients, high-risk groups, and relatives who are approached with expert advice and partnerships intensification with patient associations. Anyone interested in starting a regional alliance and implementing the 4-level approach may carry it out on their own. The decision had to be made: how to begin?

We started in 2021 aiming at the sustainable community effect, which is relevant to the topic of alleviating the mental health impact of the COVID-19 pandemic. With the declaration publicly signed on our initiative by more than a dozen of capital city district mayors 18 months later (October 2022), it seems that our first activities, preceding the official beginning of the intervention (August 2021 to December 2021), were relevant to raise the awareness and build the supportive climate for normalizing depression.

### 1.4. The Location of New Activities

The city referred to is Warsaw and especially Warsaw City Center (downtown district). We are acting with the support of the capital city and district mayors, who have become allies of the district alliance. The opening of the alliance in Warsaw was held under the patronage of the European Commission Representation in Poland, and we were also a co-founder of the World Health Organization’s Pan-European Mental Health Coalition at the time of the opening ceremony. Within this framework, we are developing models of leadership in the field of public mental health, sharing the Warsaw experience with WHO. We sensitize residents and institutions to the situation of people at risk of and experiencing depression. To this end, we are creating an inner-city alliance against depression, which is also intended to help prevent suicidal behavior. Formal and informal allies in this alliance become those individuals, institutions, and media interested in Warsaw who want to promote the three thoughts: depression can affect anyone; depression is a real disorder; depression can be effectively treated. Why Warsaw? As the capital city of Poland, this particular city can radiate the ideas implemented here to other parts of Poland and even Eastern Europe.

### 1.5. The Partner Description and Role

Warsaw City Center Alliance Against Depression (hashtagged #AliansPrzeciwDepresji in social media since the beginning) began with a series of opening events. The mapping and awareness-raising activities to destigmatize depression locally were held by the action research unit at the Maria Grzegorzewska University in Warsaw between August and November 2021. Warsaw City Center is a location of specific recognizability around Poland. In addition to organizations and entities focused on the local level, a lot of central institutions have their headquarters and offices located here, and the City Center of Warsaw is also home to recognizable landmarks, parks, and monuments, attracting visitors and tourists. It may be nationally the most recognizable district of Poland’s capital. Such a location requires a specific approach. To reach the groups within the community, we decided to focus on local, metropolitan, and mass media work from the beginning.

## 2. The First Implementation Activities and Goals

### 2.1. Forming the Alliance and Ongoingly Reflecting Its Growth on the Website

First, the grass-roots website (hosted for free on a popular Web service) was created with an invitation to form together a local alliance against depression (https://sites.google.com/view/aliansprzeciwdepresji, accessed on 15 February 2023) with later added the .org suffix (https://www.aliansprzeciwdepresji.org, accessed on 15 February 2023) and it was sent out to local stakeholders in the Warsaw’s City Center (The invitation text was as follows: “If you like, join the Warsaw City Center Alliance Against Depression. In the years 2021–2024, we are conducting research in action in the Warsaw City Center to sensitize residents and institutions to the situation of people at risk of depression. For this purpose, we are creating an alliance against depression in the Warsaw downtown area to also help prevent suicidal behavior. The formal and informal allies in this alliance are all people, institutions, and media interested in Warsaw City Center who want to promote three ideas: depression can affect anyone; depression is a real disease; depression can be treated. The alliance has no legal status, and the allies act at their own pace, at their own discretion, and do not make any legally binding commitments to each other or to other parties. The list of allies will appear in the first year of the initiative and will be constantly updated. You can also support the alliance without appearing on the list. We invite all interested participants to contact me and get involved! Piotr Toczyski, M.Psych., Ph.D. (bio), Head of Media and Communication Department at M. Grzegorzewska University in Warsaw. E-mail: ptoczyski@aps.edu.pl. Please watch the 1-minute film about our method (courtesy of the Alliance Against Depression (AAD) Coordination Centre established at the Western Australia Primary Health Alliance in Perth, whose role is to support interested Western Australian communities in building their own local alliances against depression and share what they learn with the European alliance against depression and its global network partners): https://www.youtube.com/watch?v=ZOtTHl4-bgM&ab_channel=WAPrimaryHealthAlliance” (the above link accessed on 15 February 2023). Around twenty local institutions responded to the call and actively engaged in the creation of the alliance emerging in the City Center. Some of them are already mentioning their affiliation with the Alliance on their websites and social media, becoming early ambassadors of this initiative on a local and national scale (See, for example (1) “Zobacz…. JESTEM’’ Foundation website: http://zobaczjestem.pl/srodmiejskiego-aliansu-na-rzecz-przeciwdzialania-depresji-zapraszamy-do-dyskusji (accessed on 15 February 2023); (2) eFKropka Foundation Facebook fanpage: https://www.facebook.com/permalink.php?story_fbid=4385034501562575&id=399188573480541 (accessed on 15 February 2023); (3) Mr. Paweł Kwiecień (EFPA EuroPsy-certified psychologist) website: https://www.pawelkwiecien.pl/artykuly/srodmiejski-alians-przeciw-depresji (accessed on 15 February 2023). The Warsaw City Center Alliance Against Depression was also mentioned during some events, such as (4) Transition Group’s online conference on “Employee’s psychological well-being as an essential asset to your organization” (in the closing remarks by the conference moderator Ms. Anna Pfejfer-Buczek)).

### 2.2. Launching the Alliance

While Warsaw’s City Center is often chosen as a venue for public events, they are not always targeted at the local community. Due to this specificity of Downtown Warsaw and with the purpose of reaching the local stakeholders in mind, we decided to replace a single kick-off meeting and opening ceremony with several simultaneous methods to launch the Warsaw Alliance’s activities more proactively. As a result, five public events were planned to mark a two-month awareness-raising period built around World Suicide Prevention Day (10 September 2021), European Depression Day (3 October 2021), and World Mental Health Day (10 October 2021)—occasions that gave rise to public discourse about depression and announced the creation of the alliance. The idea of the Warsaw City Center Alliance Against Depression was introduced to the general public and local stakeholders on four dates (28 August 2021, 8 September 2021, 24 September 2021, and 12 October 2021), with one more event planned for 26 October 2021, but at that time moved to another location because of the partial lockdown regulations. Because of the COVID-19 pandemic and ever-changing regulations on public gatherings in this period, it was decided to offer mostly media, online, and hybrid events to the local community of Warsaw.

#### 2.2.1. Beginning of Metropolitan Media Presence

A total of 10 days before the first official launching event, the idea of an “alliance against depression” was presented, and the creation of Warsaw’s City Center Alliance was announced in the media, namely by the top metropolitan radio station Tok FM. Almost an hour-long live discussion was broadcasted at 9 p.m. on 28 August 2021, and then archived as a podcast entitled “Alliance against depression. How can a project from Warsaw’s City Center inspire other communities in Poland?” with the lead highlighting that “Everyone can fight depression or support their loved ones: neighbors, students, teachers, employers, librarians, pub and cinema owners. How?” (available at https://audycje.tokfm.pl/podcast/110757,Alians-przeciw-depresji-Jak-projekt-ze-Srodmiescia-moze-inspirowac-inne-spolecznosci-w-Polsce (accessed on 15 February 2023)).

Three local experts, suicidologist Ms. Marta Wrona, psychiatrist Wiktor Buczek M.D., and public health campaigner Mr. Mateusz Biernat, joined Ms. Małgorzata Wołczyńska (Radio Tok FM host and editor of broadcast programs focused on health and social issues (See: https://audycje.tokfm.pl/prowadzacy/152,Malgorzata-Wolczynska (accessed on 15 February 2023)) and Dr. Piotr Toczyski (Maria Grzegorzewska University and EAAD-Best) to discuss local alliances against depression as a method worth spreading throughout Poland. We received positive feedback from the local stakeholders, proving that this media teaser has contributed to raising the profile of the alliance as a method both locally and nationally and has attracted the attention of other communities in Poland.

#### 2.2.2. Remote Public Kick-Off & Mapping Event

The week starting on 6 September 2021 (Monday) and ending on 12 September 2021 (Sunday) was the awareness-raising week around the issues of well-being and suicidality in Poland. We used this opportunity to host the first 90 min opening meeting of the Warsaw City Center Alliance Against Depression. It took place on 8 September 2021 at noon, and due to COVID-related restrictions, which would restrain representatives of some of the interested parties from participating in an in-person meeting, it was held online on Zoom. Thanks to its remote form, the meeting was open to all interested persons and institutions (if registered prior to the event) and involved the active participation of different local groups, organizations, and other entities’ representatives, who discussed why depression was so difficult to be talked about locally and what could be performed to enable more vivid and destigmatizing public communication on depression at the local level.

The invited speaker—Mr. Maciej Sopyło from the civil society Digital Challenges Group headquartered in Warsaw’s City Center—briefly presented the results of a recent (2020/2021) research [16] on the dynamics of internal communication between local stakeholders and influencers within local communities in Poland (Other speakers, who discussed challenges as well as ideas for communicating depression locally, included the following: Ms. Katarzyna Chotkowska (psychologist from eFKropka Foundation registered in Warsaw’s City Center and co-organizing the Mental Health Congress), Ms. Jolanta Palma (suicidologist from the Social Welfare Center, co-manager of district mayor’s telephone helpline for residents of Warsaw’s City Centre), Ms. Agnieszka Pawłowska-Fryckowska (from the OPTA Association which has been active in Warsaw’s City Center for 25 years and has ran psychological support programs co-financed by the City of Warsaw), Ms. Małgorzata Libman-Sokołowska (psychotherapist working with youth, families and adults, school psychologist from School Complex no 22 in Warsaw), Ms. Martyna Florczak (president of the Independent Students’ Association at the University of Warsaw—the biggest students’ union in Warsaw’s City Center), Ms. Anna Hasterok (psychotherapist associated with the nationwide initiative Psychologists and Psychotherapists for Society), and Ms. Maryanne Chodkowski (director of the prestigious Bednarska Elementary School) accompanied by Ms. Anna Plisiecka (psychologist from the same school located in Warsaw’s City Center). The debate was moderated by Ms. Marta Smagowicz (for many years associated with Warsaw-based NGOs, co-founder of the local alliance against depression), Mr. Marcin Grudzień (psychotherapist from Honeycomb Center for Psychotherapy and Support for Families in Warsaw’s City Center), and Dr. Piotr Toczyski (Maria Grzegorzewska University, EAAD-Best)). To briefly share the main takeaways from the meeting, the invited speakers concluded that although depression seemed to be destigmatized in the national public discourse, locally, it was still a very difficult topic to address. Alliance members agreed on the idea of supplementing this public kick-off meeting with a summary of the discussion and sharing it widely with the media. As a result, a five-page memo summarizing the meeting was prepared (in Polish) and then proofread and authorized by the speakers (to build a culture of trust). It was made available on our local website and on one of our LinkedIn profiles (https://www.linkedin.com/feed/update/urn:li:activity:6853029636104867840—viewed ca. 1000 times, accessed on 15 February 2023) tagged with #AllianceAgainstDepression #Warsaw #WarsawCityCenter #MentalHealthDay2021 and #WorldMentalHealthDay in English and Polish language versions. It was also sent out to all locally involved stakeholders.

#### 2.2.3. Live Radio Discussion with Metropolitan Radio Listeners

Soon after the kick-off meeting, on 14 September 2021 at 8 p.m., Warsaw City Center Alliance Against Depression went live on air again on the top Warsaw radio station. It was an 80 min live discussion within the evening “Radio Tok FM Microphone” format, which involved participation and interventions from listeners who shared their lived experience of depression and engaged in empathetic discussions with the radio host, Ms. Małgorzata Wołczyńska. Ms. Maryanne Chodkowski, the principal of Bednarska Primary School in Warsaw, was invited as a dial-in guest on the radio program, because the model of psychological aid and counseling offered in the school managed by Ms. Chodkowski was also discussed. The live broadcast was supported by EFPA EuroPsy certified psychologist Dr. Piotr Toczyski (Maria Grzegorzewska University and EAAD-Best), who was present in the studio throughout the program to debrief the audience and deliver expert insights on destigmatizing depression.

The broadcast was entitled “A reason to feel ashamed, a problem in the papers, or maybe a topic like any other? Is depression still a taboo in Poland?” and after the live streaming was made available to listeners as a podcast accessible on the website of the Tok FM radio (https://audycje.tokfm.pl/podcast/111508,Powod-do-wstydu-skaza-w-papierach-a-moze-temat-jak-kazdy-inny-Czy-depresja-to-jeszcze-w-Polsce-tabu, accessed on 15 February 2023).

#### 2.2.4. Local Experts’ Debate in the Factual Culture House

On Tuesday, 12 October 2021 at 5 p.m., the public event mapping local activities and programs addressing depression in Warsaw, entitled “Addressing depression among youth-what forms of support are effective?”, was chaired by Ms. Marta Smagowicz. Our guests were Ms. Aldona Żejmo-Kudelska from the Drama Way Foundation, involved in drama-based mental health education programs for schools, Ms. Matylda Przepiórkowska from the center for prevention and therapy of children and youth “Perspektywa”, Ms. Renata Chronowska from “Zobacz…. JESTEM” Foundation, who specializes in depression and suicide prevention, and Ms. Małgorzata Libman-Sokołowska, a psychologist and psychotherapist who works with adolescents and families in high school and at a community care center. The meeting was addressed mainly to teachers, parents, and professionals working with youth, but also to the general public. Both the local Warsaw City Center Alliance Against Depression and the European organization EAAD were mentioned in the invitation.

The City of Warsaw co-funds the Factual Culture House and its activities for the local community as well as some of the activities by organizations and institutions represented by our guest speakers, which are targeted at local youth, parents, and health professionals. Thus the official logo of the capital city of Warsaw—the Warsaw Mermaid—was visible in the background for the whole duration of the live event, helping to build the local attachment of the alliance.

The event was met with great interest from the audience. Through live streaming (via Facebook Live (https://www.facebook.com/events/977498686140574, accessed on 15 February 2023), the broadcast attracted over 500 visitors within the first three hours after the 1 h meeting.

Ms. Renata Chronowska brought leaflets about depression and suicide prevention co-funded by the City of Warsaw and distributed them among participants. Additionally, public health expert Ms. Magdalena Dąbkowska introduced the topic of possible gender differences in addressing depression in youth.

#### 2.2.5. Theater Performance on Depression

Before and during the debate in Factual Culture House, the next event to take place in the same space was teased by one of the panelists. What was still planned as the finale of the opening series of events was a theater performance about depression, which would be an artistic, drama-based activity engaging the audience.

This closing, artistic part of the multi-piece launch of the Warsaw City Center Alliance Against Depression had to be postponed until the rules of the participatory audience involvement during the pandemic (e.g., regarding social distancing, wearing masks) got clarified, and some of the internal institutional bans were lifted. The theater performance was about to take place on 26 October 2021 at 10 a.m. in the Factual Culture House in the City Center district of Warsaw, but it had to be moved to another location: one of the Warsaw schools, where school psychologists gathered with their pupils and students and other interested persons (ca. 100 attendees).

Our artistic part of the Warsaw City Center Alliance Against Depression opening series of events is based on applied theater as an intervention in well-being, the approach worked out at the University of Exeter, combining drama and performing arts with medical and health sciences, public health and health services (https://impact.ref.ac.uk/casestudies/CaseStudy.aspx?Id=39125, accessed on 15 February 2023). It is described as art having a proven impact on the improvement in community understanding of mental health, professional development for medics and teachers, as well as for training in applied and community theater. It is also said to effectively engage audiences and communities, professional or geographical, in narratives of illness, disease, disorder and stress. The story of a boy experiencing depression is derived from previous work entitled ‘On the Edge’, which is said to provide ‘a workable, reproducible and adaptable educational programme, supported by materials, evidence and experience’ [17]. 

#### 2.2.6. Other Activities

The alliance against depression was mentioned in Press (November–December 2021), a prestigious magazine for journalists and media makers, within the context of a working meeting of the alliance in Warsaw Bookstore Korekty in a central location of the city. The aim was to destigmatize depression among media gatekeepers and raise their awareness of the alliance as a way of working locally. Two media managers from top Warsaw-based media were present at the meeting: Ms. Maria Czarkowska (Gazeta Wyborcza daily) and Dr. Anna Gumkowska (TVN Discovery television and online group), discussing alliance and #iFightDepression tool with Dr. Piotr Toczyski (EAAD-Best), all photographed by Mr. Maksymilian Rigamonti, internationally awarded artist.

Simultaneously some dissemination activities were undertaken in order to build awareness of the method in Poland. Within the panel “Problems of psychological and psychiatric care in Poland and potential solutions”, Dr. Piotr Toczyski presented a paper, “Is there already a place in Poland for local alliances and online tools against depression?” (23 October 2021), and discussed with other panelists on the “Access to psychological and psychiatric care in Poland. The impact of the COVID-19 pandemic on psychological well-being” (24 October 2021). It was in real-time noted publicly especially by the national union of students (NZS) and the national union of doctoral students (KRD) on their Twitter accounts and disseminated.

## 3. Conclusions

There are not yet many metrics available to share, concerning the beginning of work on the alliance against depression in Warsaw. The main metric is the number of public events held. Five of the events attracted up to 1000 views online each. The alliance set in Warsaw began to grow organically, but the bottom-up approach does not seem to work without stimuli from the coordinating bodies. However, the mentions of the alliance and awareness-raising messages about depression in the local city center mayor’s newspaper allowed reaching ca. 30,000 readers with each piece of content about the endeavor. There are 100,000 inhabitants in the district, so we may assume that almost every third inhabitant of Warsaw city center district was exposed to the message about the alliance against depression via the mayor’s print bulletin.

We now can say that there would hardly be any local or hyperlocal interest in the topic of depression without such an alliance. It seems that local alliances against depression bring a new identity to public health issues at the hyperlocal level, and for this reason, they indeed need public events and media presence. The media, in the participatory action research framework, become part of the action; thus, they become action media.

There are four lessons we learned during the preliminary alliance-building process. The first lesson is that the local alliance in the urban local context Poland can be launched quickly, even impromptu. The informal Warsaw City Center Alliance Against Depression has been officially set up in the central district of the capital city of Poland within a few weeks. In the metropolitan context of Eastern Europe, the action may be faster than it would be in smaller communities, where the stigma burden is higher.

The second lesson is that the alliance since the beginning may be versatilely embedded in several local outlets (public discussions, media, engaged theater performances, digital channels, and hashtagged online spaces such as #AliansPrzeciwDepresji). Such a mix of media may be beneficial for public visibility and may raise the attractiveness of the alliance.

The third lesson concerns the alliance impact indicators. In our first steps, we focused on the fast track of engaging the local community toward destigmatizing depression locally. Some preliminary evidence of engagement has been included in the current short report. The visibility and community engagement opened the gates to more serious officials’ engagement, which resulted in signing the declaration “Warsaw Against Depression” 18 months later, during World Mental Health Day (10 October 2022) (See English version on YouTube with more than 200 views, https://www.youtube.com/watch?v=xkzuygX5iU4 (accessed on 15 February 2023), whereas the Facebook version in Polish has been viewed 2400 times). This can be further quantified; 15 out of 18 district mayors (or their representatives) in Warsaw publicly signed the declaration.

The fourth lesson is that animating social innovation with an aspect of creative endeavors is indeed possible informally and can be prolonged without the need for considerable additional resources. It can be achieved as long as the alliance concerns the way of working together as a process rather than achieving specific output targets. None of the local participants of the opening alliance was funded in any way. Their involvement has been truly volunteer. However, the action research funding from the European Commission concerned the facilitators from the local public university.

Could the initiative be duplicated in comparable settings or in capital cities and central communities in almost any European context? At this point, we do not know, but we encourage other cities to try it. As it was mentioned at the beginning, we drew our ideas from a method based on the European Alliance Against Depression (EAAD), which is the way the community may work together toward destigmatizing depression on a local and hyperlocal level. We creatively adjust the EAAD ideas to the local settings. As some of the activities in this endeavor were co-funded by the 3rd Health Programme of the European Commission under its 2020 edition, we pay readers attention to the fact that within EAAD method any private or public small grants (with categorically no pharmaceutical industry involvement due to the conflict of interest) may thus be useful to begin other alliances. The current brief report may serve as an idea repository to be showcased and adapted in the case of any community that may want to launch its own informal alliance in contact with EAAD.

## Data Availability

All data publicly available in the aforementioned internet links.
